# Hormone treatment as first line therapy is safe and relieves pelvic pain in women with bowel endometriosis

**DOI:** 10.31744/einstein_journal/2019AO4583

**Published:** 2019-04-25

**Authors:** Marina Paula Andres, Renata Franco Pimentel Mendes, Camila Hernandes, Sérgio Eduardo Alonso Araújo, Sérgio Podgaec

**Affiliations:** 1 Hospital das Clínicas, Faculdade de Medicina, Universidade de São Paulo, São Paulo, SP, Brazil.; 2 Universidade de São Paulo, São Paulo, SP, Brazil.; 3 Hospital Israelita Albert Einstein, São Paulo, SP, Brazil.

**Keywords:** Endometriosis/therapy, Ultrasonography, Sigmoid diseases, Hormones/therapeutic use, Contraceptives, oral, combined, Progestins, Pelvic pain, Endometriose/terapia, Ultrassonografia, Doenças do colo sigmoide, Hormônios/uso terapêutico, Anticoncepcionais orais combinados, Progestinas, Dor pélvica

## Abstract

**Objective:**

To evaluate clinical features and complications in patients with bowel endometriosis submitted to hormonal therapy.

**Methods:**

Retrospective study based on data extracted from medical records of 238 women with recto-sigmoid endometriosis treated between May 2010 and May 2016.

**Results:**

Over the course of follow-up, 143 (60.1%) women remained in medical treatment while 95 (39.9%) presented with worsening of pain symptoms or intestinal lesion growth (failure of medical treatment group), with surgical resection performed in 54 cases. Women in the Medical Treatment Group were older (40.5±5.1 years *versus* 37.3±5.8 years; p<0.0001) and had smaller recto sigmoid lesions (2.1±1.9 *versus* 3.1±2.2; p=0.008) compared to those who had failed to respond to medical treatment. Similar significant reduction in pain scores for dysmenorrhea, chronic pelvic pain, cyclic dyschezia and dysuria was observed in both groups; however greater reduction in pain scores for dyspareunia was noted in the Surgical Group. Subjective improvement in pain symptoms was also similar between groups (100% *versus* 98.2%; p=0.18). Major complications rates were higher in the Surgical Group (9.2% *versus* 0.6%; p=0.001).

**Conclusion:**

Patients with recto-sigmoid endometriosis who failed to respond to medical treatment were younger and had larger intestinal lesions. Hormonal therapy was equally efficient in improving pain symptoms other than dyspareunia compared to surgery, and was associated with lower complication rates in women with recto-sigmoid endometriosis. Medical treatment should be offered as a first-line therapy for patients with bowel endometriosis. Surgical treatment should be reserved for patients with pain symptoms unresponsive to hormonal therapy, lesion growth or suspected intestinal subocclusion.

## RESUMO

**Objetivo:**

Avaliar características clínicas e complicações em pacientes com endometriose intestinal submetidos ao tratamento hormonal.

**Métodos:**

Dados de prontuários de 238 pacientes com endometriose de retossigmoide tratadas entre maio de 2010 e maio de 2016 foram coletados para este estudo retrospectivo.

**Resultados:**

Durante o período de acompanhamento, 143 (60,1%) mulheres mantiveram tratamento clínico, enquanto 95 (39,9%) tiveram piora dos sintomas de dor ou aumento da lesão intestinal (grupo falha de tratamento clínico), sendo 54 submetidas ao tratamento cirúrgico. As mulheres no Grupo Tratamento Clínico eram mais velhas (40,5±5,1 anos *versus* 37,3±5,8 anos; p<0,0001) e tinham lesões intestinais menores (2,1±1,9 *versus* 3,1±2,2; p=0,008) em comparação ao grupo falha de tratamento clínico. Redução significativa e semelhante do escore de dor na dismenorreia, dor pélvica crônica, disquezia cíclica e disúria cíclica foi observada nos Grupos Tratamento Clínico e Cirúrgico. Dispareunia, no entato, teve uma redução maior no Grupo Cirurgia. A redução subjetiva dos sintomas dolorosos também foi semelhante entre os Grupos Clínico e Cirúrgico (100% *versus* 98,2%; p=0,18). O Grupo Tratamento Cirúrgico foi relacionado a uma maior taxa de complicações graves (9,2% *versus* 0,6%; p=0,001) em comparação ao Grupo Tratamento Clínico.

**Conclusão:**

Falha no tratamento clínico em pacientes com endometriose de retossigmoide foi observada em mulheres mais jovens que tinham lesões intestinais maiores. O tratamento clínico hormonal foi igualmente eficaz na melhora dos sintomas de dor, exceto dispareunia, em comparação ao tratamento cirúrgico em mulheres com endometriose intestinal, mas com menor taxa de complicações. O tratamento clínico deve ser oferecido como primeira opção em pacientes com endometriose intestinal, enquanto o tratamento cirúrgico deve ser reservado para pacientes sem melhora nos sintomas de dor com tratamento hormonal, progressão das lesões ou suspeita de suboclusão intestinal.

## INTRODUCTION

Endometriosis is the presence of endometrial tissue outside the uterus. The estimated prevalence of the disease ranges from 10% to 15% in child-bearing age women, and may amount to 70% and 48% in patients with chronic pelvic pain and infertility respectively.^(^ ^1^ ^)^ Three different types of endometriosis have been described: deep endometriosis, peritoneal endometriosis and ovarian endometrioma.^(^ ^2^ ^,^ ^3^ ^)^ Deep endometriosis accounts for almost half of endometriosis cases, with bowel involvement in 50% of them.^(^ ^4^ ^,^ ^5^ ^)^

The clinical suspicion of endometriosis is based on history and physical examination.^(^ ^6^ ^,^ ^7^ ^)^ The diagnostic imaging modality of choice is transvaginal ultrasound (TVUS) with bowel preparation, given its low cost, ease access and high accuracy. Reported sensitivity and specificity for rectosigmoid lesions are 98.1% and 100%, respectively.^(^ ^5^ ^)^ Bowel preparation prior to TVUS is aimed at elimination of bowel contents and reduction of imaging artifacts generated from rectal gases and fecal matter.^(^ ^8^ ^,^ ^9^ ^)^

Once the suspicion of endometriosis has been confirmed, clinical or surgical treatment may be selected. However, adequate treatment is complex and controversial,^(^ ^10^ ^,^ ^11^ ^)^ given the heterogeneous nature of the disease and the different clinical conditions presented by affected patients.^(^ ^12^ ^,^ ^13^ ^)^

Clinical management of endometriosis is based on menstrual cycle reduction. Progestogens and combined contraceptives are particularly recommended and, according to current evidence and gynecological society guidelines, the different hormone treatments are equally effective.^(^ ^13^ ^-^ ^22^ ^)^ Treatment choice should be based on patient's pregnancy desire and clinical characteristics, and location of endometriotic lesions.^(^ ^23^ ^)^ Clinical management aims to relieve pain symptoms and quality of life, as well as lesion stabilization.^(^ ^3^ ^)^ However, it is an obstacle to women who want to get pregnant.^(^ ^13^ ^)^

Surgery is required for cases of intestinal endometriosis refractory to medical treatment, and cases with obstructive lesions or intestinal obstruction.^(^ ^3^ ^)^ Resection of endometriosis implants has been shown to improve pelvic pain and patient's quality of life, and to decrease disease recurrence rates.^(^ ^22^ ^-^ ^25^ ^)^ However, major complications associated with surgical treatment of bowel endometriosis were reported in 6.3% of cases, including thrombosis, infection, hemorrhage, anastomotic leakage and injured bowel, ureter, bladder and large vessels during surgical procedure.^(^ ^26^ ^,^ ^27^ ^)^

## OBJECTIVE

To compare pain relief, complication rates and efficacy of treatment in patients with rectosigmoid endometriosis submitted to clinical or surgical management, and to evaluate medical treatment as a first-line therapy.

## METHODS

A retrospective cohort study was carried out based on data collected from medical records of women with rectosigmoid endometriosis treated between May 2010 and May 2016.

This study was approved by Research Ethics Committee of *Hospital das Clínicas, Faculdade de Medicina, Universidade de São Paulo*, protocol no. 1.631.417, CAAE: 57077816.0.0000.0068. Given the retrospective design, this study was exempt from informed patient consent.

Rectosigmoid endometriosis was defined as the presence of intestinal lesions larger than 5mm in TVUS with bowel preparation. Patients with a history of surgical treatment for endometriosis, inflammatory pelvic disease or inaccessible or incomplete data were excluded.

Following anamnesis and complete gynecological examination, all patients with suspected endometriosis were submitted to TVUS by a radiologist. Whenever recto-sigmoid lesions were diagnosed, hormonal therapy with combined contraceptives or continued use of progestogens was indicated, except for patients expressing desire to get pregnant. Surgery was promptly indicated in cases of ureteral, appendicular and ileal or recto-sigmoid endometriosis with partial bowel occlusion.

Patients included in the analysis were followed for at least 6 months, then submitted to clinical and imaging reassessment. Lack of improvement in symptoms or lesion growth was defined as failure of medical treatment.

Patients were assessed for dysmenorrhea, acyclic pelvic pain, intense dyspareunia and intestinal and urinary pain symptoms associated to cycles, using a zero-to-ten visual analog scale (VAS) for pain. Other parameters analyzed were type of treatment and complications occurring over the course of follow-up. Symptoms reported by patients under exclusive medical treatment (Clinical Group) and patients submitted to surgical treatment (Surgical Group) were also compared. Patients were assessed at first and 6-month follow-up visits, or preoperatively and within 3 months of surgery (Clinical and Surgical Group, respectively).

Sample normality was investigated using the Komolgorov-Smirnov test. Statistical analysis was performed using the software Statplus for Mac (version 5.8). Categorical and continuous variables were analyzed using χ^2^ test or Fisher's exact test, and the Student's *t* test or Mann-Whitney test, respectively. The level of significance was set at 5% (p<0.05).

## RESULTS

A total of 2,275 patients were seen at the Endometriosis Clinic between May 2010 and May 2016, and 358 of them were diagnosed with recto-sigmoid endometriosis on TVUS with bowel preparation. A total of 120 patients were excluded, as follows: 63 (17.6%) due insufficient data in the medical records, 37 (10.3%) due to incomplete follow-up, 15 (4.2%) due to previous surgical treatment, and 5 due to direct referral for surgery (4 with appendicular endometriosis, and 1 with ileal endometriosis). The final sample comprised 238 patients.

**Figure 1:**
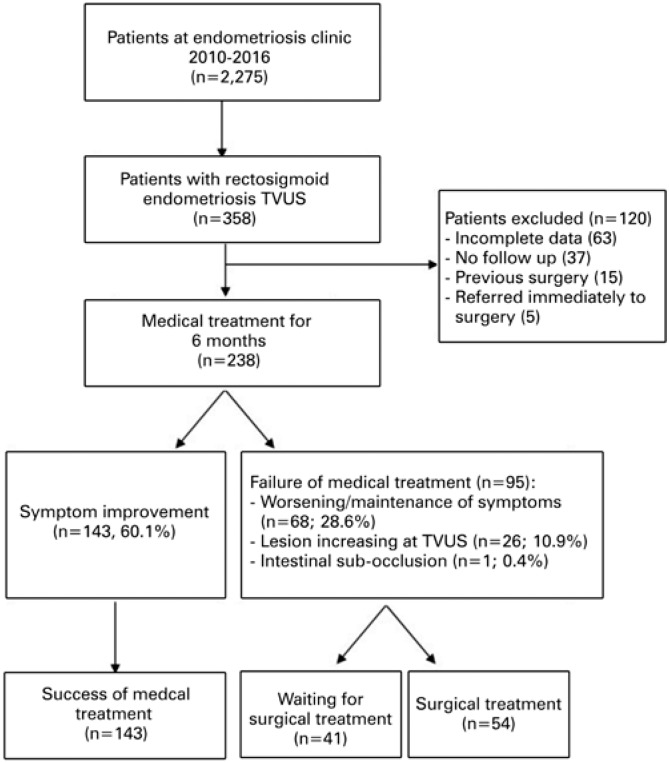
Patient selection and inclusion flow TVUS: transvaginal ultrasound.

Treatment flow is shown in figure 1. After 6 months, 143 (60.1%) patients reported improvement in pain symptoms and did not require surgery, while 95 (39.9%) were referred for surgical treatment due to worsening or persistence of pain symptoms (n=68; 28.6%), growth of endometriosis lesions (n=26; 10.9%) or symptoms of bowel partial occlusion (n=1; 0.4%).

Over the course of the study period, 54 patients underwent surgical treatment for endometriosis (Surgical Group), 41 were referred for surgery and 143 remained under exclusive medical treatment (Clinical Group). Patients with surgical indication (failure of medical treatment; n=95) were grouped for analytic purposes.

Patients responding to medical treatment were older compared to non-responsive patients (40.5±5.1 years *versus* 37.3±5.8 years; p<0.0001). Mean patient follow-up time was 19.9±13.6 months and 20.5±15.5 months (responsive and non-responsive patients respectively). Clinical parameters are shown in table 1. Patients who failed to respond to medical treatment had higher VAS scores for dysmenorrhea, dyspareunia, chronic pelvic pain and cyclic dysuria and dyschezia at the time of first medical visit compared to responsive patients.

**Table 1:** Clinical characteristics of patients diagnosed with recto-sigmoid endometriosis per group (success or failure of medical treatment)

Characteristics	Medical treatment		p value
	Success	Failure	
	(n=143)	(n=95)	
Age, years	40.5±5.1	37.3±5.8	0.0001*
BMI, kg/m^2^	27.4±5.2	26.8±4.9	0.566*
Follow-up, months	19.9±13.6	20.5±15.5	0.72*
Infertility	66 (46.2)	54 (56.8)	0.13†
Dysmenorrhea, VAS	4.9±4.0	6.2±4.3	0.02*
Dyspareunia, VAS	3.6±3.9	5.1±3.9	0.004*
Chronic pelvic pain, VAS	3.6±3.7	5.1±3.6	0.002*
Dysuria, VAS	0.7±1.7	2.0±3.5	0.0001*
Dyschezia, VAS	2.0±2.9	3.6±3.8	0.0007*

Results expressed as mean±standard deviation or n (%).

* Student *t* test;

† χ^2^ test.

BMI: body mass index; VAS: visual analogue scale.

Sonographic TVUS features of recto-sigmoid lesions are summarized in table 2. Patients who failed to respond to medical treatment had larger recto-sigmoid lesions (3.1±2.2 *versus* 2.1±1.9; p=0.008) and greater compromised intestinal loop circumference (28.8±10.6 *versus* 25.0±11.4; p=0.02) compared to responsive patients (3.1±2.2 *versus* 2.1±1.9; p=0.008). Medical treatment was offered to all women and varied according to respective profile and complaints. Oral progestogen (50.5%), combined contraceptives (18.6%), analgesics (17.6%), medroxyprogesterone injection (8.0%), levonorgestrel-releasing intrauterine device (4.3%) and GnRH analogues (1.1%) were the most commonly prescribed treatments.

**Table 2:** Sonographic (transvaginal ultrasonography with intestinal preparation) features of recto-sigmoid endometriotic lesions per group (success or failure of medical treatment)

Recto-sigmoid lesion features		Medical treatment		p value
		Success (n=143)	Failure (n=95)	
Larger diameter, cm		2.1±1.9	3.1±2.2	0.008*
Distance from anal verge, cm		11.2±2.9	11.0±2.8	0.66*
Compromised circumference, %		25.0±11.4	28.8±10.6	0.02*
Affected layer				0.16†
	Serosa	14 (9.8)	9 (9.5)	
	Muscularis	96 (65.7)	58 (61.1)	
	Mucosa	13 (9.1)	18 (18.9)	
	Not reported	20 (14.0)	10 (10.5)	

Results expressed as mean±standard deviation or n (%).

* Student *t* test;

† χ^2^ test.

All endometriotic lesions were surgically resected in the surgical group. Recto-sigmoid endometriotic lesions were treated as follows: segmental bowel resection and re-anastomosis (n=35) of multiple lesions, or lesions larger than 3cm; discoid bowel resection (n=7) in single lesions smaller than 3cm; bowel shaving in lesions affecting no deeper than external muscularis layer (n=12). Histological analysis of resected lesions confirmed the diagnosis of endometriosis in all cases.

**Figure 2:**
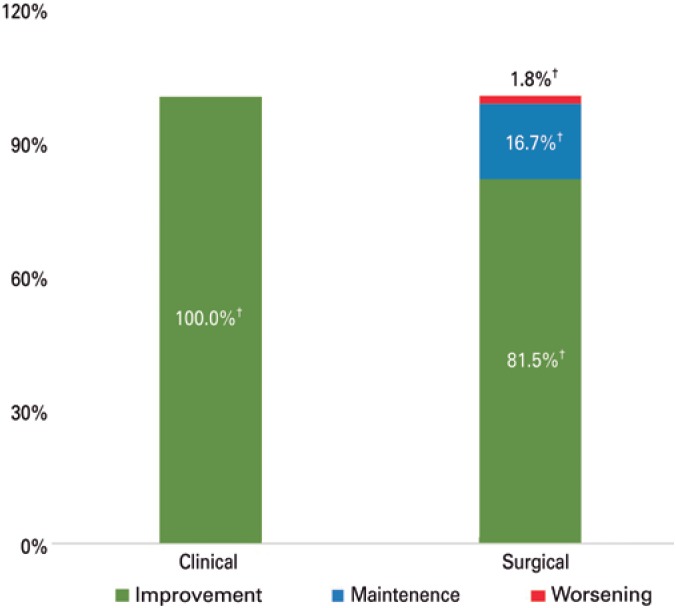
Symptom progression in patients with recto-sigmoid endometriosis submitted to medical or surgical treatment ^†^χ^2^ test.

Pain symptoms were evaluated before and after treatment (Table 3). Significant reduction in VAS scores for dysmenorrhea, dyspareunia, chronic pelvic pain, dysuria and dyschezia was observed in cases submitted to medical and surgical treatment alike. Greater reduction in VAS scores for dyspareunia was documented in the surgical compared to the Clinical Group (-3.6±4.8 *versus* 1.65±3.8; p<0.0001). However, subjective pain assessment was similar between groups, with improvement reported by most patients included in the sample (100% *versus* 98.2%; p=0.18; Figure 2).

**Table 3:** Symptoms reported by patients with recto-sigmoid endometriosis before and after medical treatment

Symptom	Medical treatment			Surgical treatment			p value†
	Before (VAS)	After (VAS)	p value*	Before (VAS)	After (VAS)	p value*	
Dysmenorrhea	4.9±4.0	2.4±3.4	<0.0001	6.1±4.4	2.1±3.5	<0.0001	0.8566
Dyspareunia	3.6±3.9	1.9±3.2	<0.0001	5.1±3.9	1.5±2.9	<0.0001	<0.0001
CPP	3.6±3.7	1.7±1.7	<0.0001	5.3±3.6	2.6±3.5	<0.0001	0.6928
Dysuria	0.7±0.1	0.1±0.8	<0.0001	2.0±3.7	0.3±1.3	0.0013	0.392
Dyschezia	2.0±2.9	0.3±1.2	<0.0001	3.7±4.1	1.4±2.9	<0.0001	0.878

Data expressed as mean±standard deviation.

* Student's *t* test;

† Mann-Whitney test comparing reduction in pain symptoms between groups submitted to medical (n=143) or surgical (n=54) treatment.

VAS: visual analogue scale; CPP: chronic pelvic pain.

Six major complications were observed during the follow-up period, as follows: Clinical Group, one patient, 0.6% (intestinal partial occlusion requiring urgent surgery); Surgical Group, five patients (9.2%; p=0.001), including incisional hernia (n=1); ileostomy wound dehiscence (n=1); vascular lesion (n=1); compartment syndrome in both legs (n=1); chronic obstipation (n=1).

## DISCUSSION

Bowel endometriosis accounts for up to 20% of endometriosis cases, with negative impacts on patient quality of life. Adequate treatment of the disease is complex and controversial.^(^ ^10^ ^,^ ^11^ ^)^ Transvaginal ultrasound has recently been introduced as a diagnostic modality for deep endometriosis, with more than 85% sensitivity and specificity reported in several studies, depending on examiner's level of expertise.^(^ ^8^ ^,^ ^9^ ^,^ ^28^ ^)^ Magnetic resonance imaging may also be used for deep endometriosis diagnosis, with similar accuracy but higher costs.^(^ ^29^ ^)^

Pain symptoms reported by 238 patients with recto-sigmoid endometriosis were compared in this study. In compliance with our treatment protocol, all patients were first submitted to medical treatment. Within 6 months, 60.1% reported improvement in pain symptoms and had no evidences of lesion growth; therefore, surgical treatment was not indicated. Failure to respond to medical treatment was more common among younger patients, and those with larger lesions. Also, at the time of this study, these patients had similar significant reduction in VAS scores for dysmenorrhea, chronic pelvic pain, dysuria and dyschezia to patients submitted to surgical treatment. However, greater reduction in VAS scores for dyspareunia was observed in surgical patients within 3 months of surgery. These findings are corroborates by other authors reporting similar results in clinical and surgical treatment.^(^ ^21^ ^,^ ^30^ ^)^ A parallel cohort study carried out in 2012 by Vercellini et al.,^(^ ^31^ ^)^ compared the outcomes of surgical and medical treatment in 154 patients with endometriosis-related deep dyspareunia. Patients reported rapid and greater improvement in pain during sexual intercourse and sexual functioning after surgical treatment. However, progressive worsening of symptoms was observed within 6 months of surgery, suggesting equivalent results of both treatments at one-year follow-up.

Complications of medical treatment were limited to 0.6% of patients (intestinal partial occlusion), with endometriotic lesion growth in 10.9%. Controversially, a 9.2% rate of major complications was observed following surgical treatment in this study (p=0.001). A recent systematic review revealed a 6.3% rate of major complications following bowel endometriosis resection, including fistula, transient urinary retention and anastomotic leakage (2.7%, 3.5%, and 0.8% of cases, respectively).^(^ ^27^ ^)^

Progestogens and combined contraceptives are often recommended as a first-line therapy for endometriosis-related pain, with high efficacy and tolerance and few adverse effects.^(^ ^21^ ^,^ ^29^ ^,^ ^30^ ^,^ ^32^ ^)^ Accordingly, oral progestogens were the most commonly prescribed medication in this study, followed by combined oral contraceptives. Only 4.3% of patients were treated with levonorgestrel-releasing intrauterine device, as reported in literature.^(^ ^31^ ^)^

Limitations of this study include retrospective design and heterogeneous medical treatment based on patient's preference and profile. Also, surgical indication based on failure to respond to medical treatment suggests these patients may have had more severe symptoms, which may have introduced a bias in the analysis of results.

## CONCLUSION

The findings of this study support the hypothesis that hormone therapy is safe and effective in relieving pain symptoms associated with bowel endometriosis. Surgical resection should be reserved for women with appendicular, ileal or cecal endometriosis, growing lesions or lack of pain improvement following 6 months of medical treatment. Patients eligible for medical treatment should be informed of potential disease persistence or even progression in spite of treatment, and should be monitored for symptoms and imaging findings. Prospective randomized trials comparing surgical and medical treatment of recto-sigmoid endometriosis should be conducted to confirm the results presented.
